# Editorial: The evolution of the brain hardware for language

**DOI:** 10.3389/fpsyg.2023.1323737

**Published:** 2023-11-01

**Authors:** Antonio Benítez-Burraco, Emiliano Zaccarella, Elliot Murphy

**Affiliations:** ^1^Department of Spanish, Linguistics, and Theory of Literature (Linguistics), University of Seville, Seville, Spain; ^2^Max Planck Institute for Human Cognitive and Brain Sciences, Leipzig, Germany; ^3^Vivian L. Smith Department of Neurosurgery, McGovern Medical School, University of Texas Health Science Center at Houston, Houston, TX, United States; ^4^Texas Institute for Restorative Neurotechnologies, University of Texas Health Science Center at Houston, Houston, TX, United States

**Keywords:** language evolution, neurolinguistics, paleoneurology, comparative neurobiology, linguisticality

Humans are endowed with a species-specific ability to learn and use languages, aka *linguisticality*. Decades of neurolinguistic research has enabled the identification of brain regions that contribute to basic syntactic operations, semantic composition, morphological unification, and complex phonological processes. These regions are mostly located in the frontal and temporal cortices, as well as selected subcortical areas, and are mostly left-lateralized in the neurotypical subject (see Hickok and Poeppel, 2004; Friederici et al., 2017; Murphy, 2020; Murphy et al., 2022, among many others). This functional language network seems to be shared by speakers of typologically-diverse languages (Malik-Moraleda et al., 2022), but also by deaf people using sign language (Trettenbrein et al., 2020). Nonetheless, the question of how these particular components evolved in the first place remains unsettled (Murphy, 2019). We still have a poor knowledge of the cytoarchitectonic, connectomic and cellular differences between modern humans and non-humans that may have helped establish these regions as the major nodes in the language network. To address these issues, an interdisciplinary approach is essential. Neuroanatomical and neurofunctional studies of the primate brain using modern imaging facilities (e.g., Friedrich et al., 2021) have significantly improved our understanding of the major differences between the human and the non-human brain. Likewise, recent paleoneurological studies have improved our comprehension of the fine-grained changes in the hominin brain that contributed to the emergence of the human language faculty (e.g., Beaudet, 2017; Bruner, 2017), including changes in brain oscillatory dynamics, seemingly enabling the development of language (e.g., Murphy and Benítez-Burraco, 2018). Some specific hypotheses have been developed about the evolutionary changes experienced by the language network, particularly by selected core components, like Broca's area (Friederici, 2023; Gallardo et al., 2023).

This Research Topic aimed to provide an update of recent investigations in the paleoneurology of language. We have brought together 4 contributions from 11 leading scholars in different research areas of interest for the questions we have highlighted above. The paper by Planer revisits a debate concerning the evolutionary links between action planning and language planning, with a focus on hierarchically structured objects. Like other scholars before him (e.g., Fujita and Fujita, 2022), Planer regards hierarchical language structures as a special case of structured actions. Accordingly, he argues that a selection toward enhanced manipulating capacities might have improved the brain machinery capable of also generating hierarchically-structured communicative representations. Friederici (2023) has recently noted that the neuroanatomical expansion of Broca's region, serving action functions in other primates, might have provided the basis for a human-specific linguistic function of this region.

The paper by Liu et al. focuses on the neural basis of a specific aspect of syntax, namely Merge, which is the operation that combines two elements to form a new constituent. Specifically, they conducted an fMRI experiment in which participants were asked to merge a third component to a two-pseudoword construction. The results suggest that this operation may be performed by the posterior inferior frontal gyrus, a component of Broca's area.

The paper by Zhang et al. reviews the neuroanatomical basis of categorical perception (i.e., the ability to perceive continuous inputs as discrete stimuli). Evolutionarily, these authors hypothesize that categorical perception could have laid the foundation for discreteness, one of the design features of human language, thus highlighting the important role played by changes to the sensorimotor systems in the evolution of human language.

Finally, the paper by Worden argues that the notable expansion of the human brain, which can be related to the evolution of modern language, was only initially driven by natural selection, with sexual selection playing a more prominent role during the last stages of human evolution.

## Author contributions

AB-B: Writing – original draft. EZ: Writing – review & editing. EM: Writing – review & editing.
